# Immunoglobulin superfamily 9 (IGSF9) is trans-activated by p53, inhibits breast cancer metastasis via FAK

**DOI:** 10.1038/s41388-022-02459-8

**Published:** 2022-09-10

**Authors:** Yaohua Li, Yiran Deng, Yannan Zhao, Wei Zhang, Si Zhang, Li Zhang, Biyun Wang, Yingying Xu, She Chen

**Affiliations:** 1 NHC Key Laboratory of Glycoconjugate Research, Department of Biochemistry and Molecular Biology, School of Basic Medical Sciences, Fudan University, 130 Dong’an Road, Xuhui District, Shanghai, 200032 China; 2 Department of Medical Oncology, Fudan University Shanghai Cancer Center, Shanghai Medical College, Fudan University, 270 Dong’an Road, Xuhui District, Shanghai, 200032 China; 3 Department of Laboratory Medicine, Shanghai General Hospital, Shanghai JiaoTong University School of Medicine, Shanghai, 200080 China

**Keywords:** Breast cancer, Focal adhesion

## Abstract

Metastasis of breast cancer represents the major reason for its poor prognosis, leading to high mortality. In breast cancer, a tumor suppressor gene *TP53* is commonly mutated. *TP53* mutation leads to an altered expression of various genes, an event that is associated with aggressive tumor and is a strong independent marker for survival. In this study, we identified a novel p53 target gene, immunoglobulin superfamily 9 (IGSF9). IGSF9 is generally down-regulated in breast cancer tissues. Loss of IGSF9 is associated with frequent metastasis and poor prognosis of breast cancer patients. Wild-type p53, but not R175H mutant, trans-activates the transcription of IGSF9 via binding to its promoter (−137 to −131 bp), inhibits epithelial-mesenchymal transition (EMT), consequently the inhibition of breast cancer cells migration and invasion. IGSF9 interacts with focal adhesion kinase (FAK) and inhibits FAK/AKT signaling activity. PND1186, FAK inhibitor, inhibits breast cancer metastasis induced by *IGSF9* knockdown in vitro and in vivo. Taken together, IGSF9 is trans-activated by p53 and inhibits breast cancer metastasis by modulating FAK/AKT signaling pathway. IGSF9 could serve as a prognostic marker and potential therapeutic target for breast cancer.

## Introduction

Breast cancer is the most common malignant cancers with high incidence and mortality in women [1], with ~2.3 million new cases and 685,000 deaths in 2020 [2]. Vast majority of breast cancer-related deaths is due to metastasis to distant organs, such as lungs [3–5]. The metastatic breast cancer (mBC) patients are mostly from recurrency after primary treatment of earlier-stage breast cancer, and partially from de novo metastasis [6]. mBC is largely an incurable disease with a 5-year survival rate around 23.8–30% [7]. Thus, understanding the underlying mechanisms of breast cancer metastasis is of paramount importance for developing effective treatment.

Tumor suppressor p53, a sequence-specific DNA binding transcription factor, regulates the expression of genes involved in a variety of cellular functions, including cell-cycle arrest, DNA repair, and apoptosis [8]. In breast cancer, p53 suppressed metastasis by upregulating an early onset breast cancer-associated gene GAS7 [9]. p53 negatively regulated the expression of a potential targeted radioiodine therapy candidate, human sodium iodide symporter [10]. *TP53* (p53) is most frequently mutated in invasive breast cancer. Mutations in *TP53* present in ~25–30% of all breast cancer cases [11], and in 80% of patients with triple-negative breast cancer (TNBC) [12]. The TNBC with mutant p53 usually have poor prognosis [13]. Wei et al. described a global map of p53 transcription-factor binding sites in the human genome [14]. Further dissect and confirm potential p53 targets will be beneficial for mBC diagnosis and treatment.

Cell adhesion molecules (CAMs) are membrane glycoprotein receptors that mediate cell–cell/matrix interaction and transduce intracellular signals for cell adhesion, migration, invasion and organ-specific metastasis [15]. CAMs include cadherins, selectins, integrins, the immunoglobulin superfamily (IgSF), and etc [15, 16]. Immunoglobulin superfamily 9 (IGSF9), first cloned in 2000 [17], is a member of the IgSF. IGSF9 contains extracellular N terminus with five Ig domains followed by two fibronectin type III domains, a signal transmembrane sequence, and a large C terminal cytoplasmic tail [18]. Previous studies reported that IGSF9 was aberrantly expressed in different cancer types, such as down-regulation in melanoma, colorectal familial adenomatous polyposis [19, 20], and upregulation in gallbladder cancer, ovarian cancer, and endometrial cancer [21–23]. Elevated IGSF9 was associated with the poor prognosis of nasopharyngeal carcinoma in Huang et al.’ study [24]. However, the molecular mechanisms underlying IGSF9 expression and cancers remain elusive, especially in breast cancer.

Focal adhesion kinase (FAK), a non-receptor tyrosine kinase, consists of N-terminal 4.1, ezrin, radixin, moesin homology domain FERM, a central catalytic tyrosine kinase domain, and a C-terminal region containing a focal-adhesion targeting domain and a proline-rich region [25, 26]. FAK acts as both a signaling kinase and cell adhesion-associated scaffold to coordinate the positional recruitment and phosphorylation of different cytoskeletal-associated proteins paxillin and p130Cas [27]. FAK could bind with integrins or growth factor receptors to control cell motility, invasion, and survival [28]. Previous studies indicated that FAK was involved in cancer development and progression [29, 30]. FAK is over-expressed and activated in invasion or metastatic breast cancer, and is associated with breast cancer progression and poor prognosis [31–33]. Wu et al.’s study showed that deletion of FAK in mammary epithelial cells suppressed tumor formation and metastasis [34].

In this study, we discovered that wild-type p53 trans-activates IGSF9 transcriptional activity by binding to its promoter. Loss of IGSF9 correlates with breast cancer metastasis and poor prognosis. IGSF9 inhibits breast cancer metastasis through FAK signaling. IGSF9/FAK axis might serve as a potential target for breast cancer treatment.

## Results

### p53 trans-activates *IGSF9* in breast cancer

**Fig. 1:**
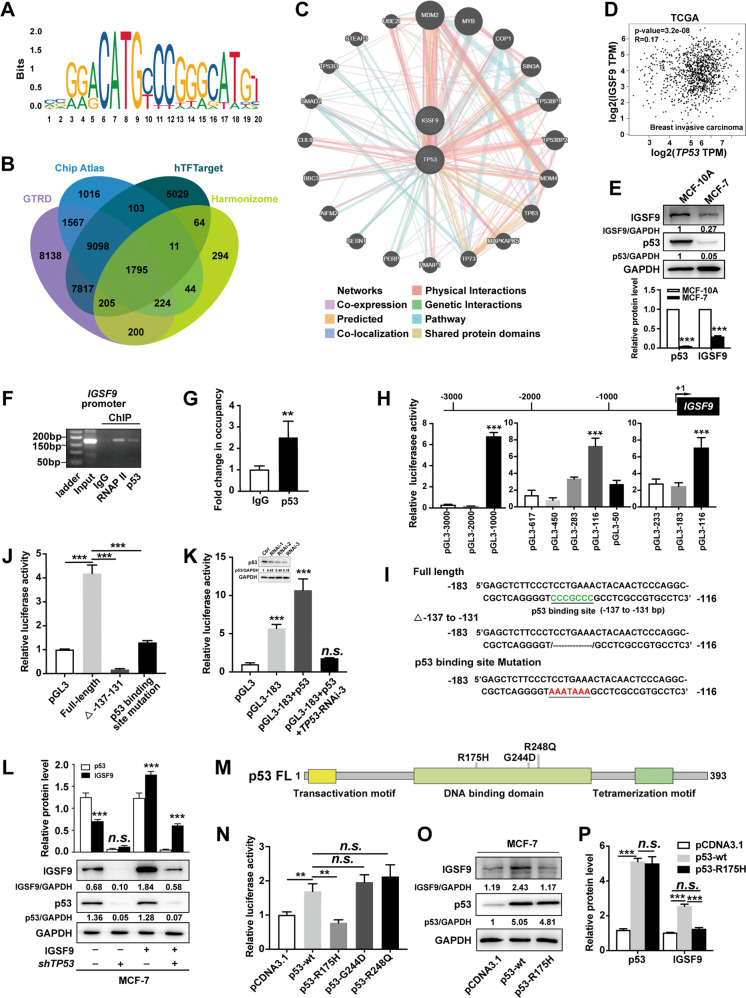
p53 trans-activates *IGSF9* in breast cancer. **A** The canonical binding site of *TP53* predicted by JASPAR is manifested by the sequence logograph. **B** Veen diagram analysis showing 1795 potential target genes of p53. **C** GeneMANIA was used to identify *IGSF9* correlating with *TP53*. 20 related genes were in the outer circle while two hub genes were in the inner circle. The color of the line shows different kinds of their correlations. **D** The positive correlation between *IGSF9* and *TP53* mRNA in breast cancer in TCGA cohort by Pearson correlation coefficient analysis. **E** The lower level of IGSF9 and p53 protein was detected in breast cancer cells. IGSF9 and p53 protein in breast cancer cells MCF-7 and normal mammary cells MCF-10A were determined by western blot. Relative intensity of IGSF9 and p53 was quantified by Image J and normalized to GAPDH. Data were represented as the mean ± SD. The Student’s *t* test was used; ***P < 0.001. p53 binds to the promoter of *IGSF9*. ChIP assay was applied to determine the binding of *IGSF9* promoter to p53. p53 antibody enriched the DNA sequence of *IGSF9* promoter. PCR (**F**) and qRT-PCR quantification (**G**) of the immunoprecipitated DNA were measured. Rabbit IgG was used as a negative control and RNA polymerase II (RNAP II) as a positive control. Values represented enrichment relative to input DNA. Data were represented as the mean ± SD. The Student’s *t* test was used; **P < 0.01. **H** −183/−116 nt region of *IGSF9* exhibited higher luciferase activity. Luciferase reporter assays were conducted to determine transcription activity of a serial of *IGSF9*-luc reporter genes ranging from −3000 to +50 bp of putative promoter. ***P < 0.001. **I** Nucleotide sequence of −183/−116 nt full length, deletion and point mutations were constructed. Transcriptional factor p53-binding sites were showed. **J** Deletion and mutations within p53 binding site (−137/−131 nt) reduced *IGSF9* promoter activity. Data were represented as the mean ± SD. The Student’s *t* test was used; ***P < 0.001. **K** p53 showed high binding capability with promoter (−183/−116 nt) of *IGSF9*. Data were represented as the mean ± SD. The Student’s *t* test was used; ***P < 0.001. **L** *TP53* knockdown decreased IGSF9 protein expression. IGSF9 was over-expressed and *TP53* was knocked down in MCF7 cells as indicated. IGSF9 and p53 protein was determined by western blot. Relative intensity of IGSF9 and p53 was normalized to GAPDH. Data were represented as the mean ± SD. The Student’s *t* test was used; ***P < 0.001. R157H, but not other mutants (G244D and R248Q) of p53 lost the transcriptional activity on *IGSF9*. Schematic diagram illustrating the protein domains of p53, and three mutation hotspots as indicated (**M**). Luciferase assay (**N**) and western blot (**O**) were conducted with MCF-7 cell. Quantification (**P**) was performed by ImageJ. Error bars denote as mean ± SD. The Student’s *t* test was used; **P < 0.01, ***P < 0.001.

p53 as a transcriptional factor regulates significant cellular activities, such as cell cycle, senescence, and apoptosis. We predicted the characteristic of optimal transcription binding sites using JASPAR database (Fig. 1A). Then Chip Atlas, hTFTarget, GTRD, and Harmonizome were used for potential target genes of p53. Venn diagram analysis revealed 1795 target genes (Fig. 1B). GO and KEGG pathway enrichment analysis indicated these potential genes mainly involved focal adhesion pathway, adherens junction, and so on (Fig. S1A, B). Metascape was also used for GO function and KEGG pathway enrichment analysis to explore the main functions and pathways of target genes (Fig. S1C). These data suggested that p53 regulated cell adhesion-related genes (CAMs). Given that IGSF9 is a member of CAMs and potential target of p53 (data from GTRD database), we further used GeneMANIA to construct the network of *TP53* and *IGSF9* to explore their interaction from different aspects (Fig. 1C).

To explore the relationship between p53 and IGSF9, we searched TCGA datasets. The positive correlation between the mRNA levels of *TP53* and *IGSF9* was observed in breast cancer patients (Pearson *R* = 0.17, *P* < 0.001) (Fig. 1D). Compared with normal mammary cell MCF-10A, lower level of p53 protein was observed, which is consistent with decreased IGSF9 level in breast cancer cell MCF-7 (Fig. 1E), suggesting the potential role of p53 in regulating *IGSF9* expression of breast cancer. The chromatin immunoprecipitation (ChIP) assay was then performed with PCR primers flanking the region of the binding site (−3000/+50 bp). The results indicated that p53 bound and enriched the promoter of *IGSF9* (Fig. 1F, G).

To further confirm the regulating role of p53 in *IGSF9* gene expression, luciferase report assay was employed to detect transcription activity of a serial of *IGSF9*-luc reporter genes ranging from −3000 to +50 bp of putative promoter. The sequences of −183/−116 nt have the highest promoter activity, and this region included p53 binding site (−137/−131 nt) (Fig. 1H). Deletion and mutation of putative p53 binding site significantly diminished *IGSF9* promoter activity (Fig. 1I, J). p53 protein significantly increased *IGSF9* transcriptional activity, which was markedly reduced by *TP53* knockdown (Fig. 1K). Consistently, *TP53* knockdown significantly decreased endogenous and exogenous IGSF9 expression in MCF-7 cells (Fig. 1L). These results indicated that p53 trans-activated *IGSF9* by binding to its promoter.

R175H, Y220C, G244D, G245S, R248Q, R249S, R273H and R282W in DNA binding domain of p53 are frequently mutated in breast cancer [9, 35]. We then made constructs bearing these mutations (Figs. 1M and S2A). Luciferase activity assays demonstrated that R175H, but not other mutation, abolished p53 trans-activated *IGSF9* promoter activity (Figs. 1N and S2B). We next examined the IGSF9 protein in MCF-7 cells, and found that over-expression of wild-type p53 significantly increased IGSF9 protein levels while R175H mutant did not change IGSF9 expression (Fig. 1O, P and Fig. S2C, D). Consistently, other p53 mutants increased IGSF9 protein similar to wild-type p53 (Fig. S2C, D). Altogether, these data confirmed that wild-type p53 directly bound to the *IGSF9* promoter and activated *IGSF9* expression, which could be abolished by R175H mutation.

### Loss of IGSF9 associates with metastasis and poor prognosis in breast cancer

**Fig. 2:**
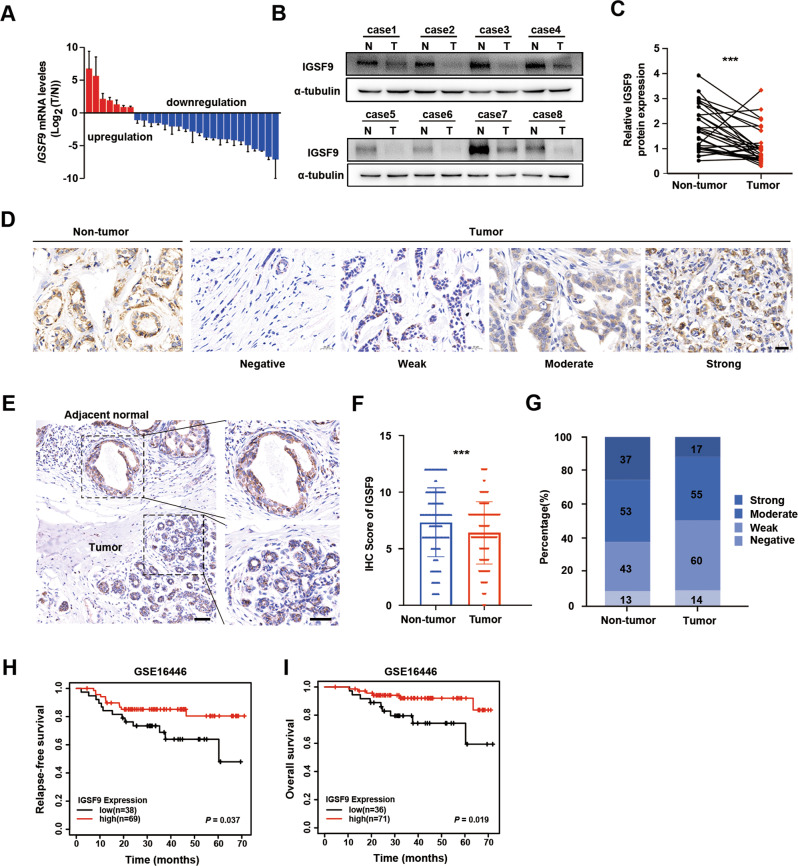
Loss of IGSF9 correlates with poor prognosis in breast cancer patients. **A** The ration (T/N) of *IGSF9* mRNA was exhibited as log(T/N). **B** IGSF9 protein level was down-regulated in breast cancer tissues. Paired breast cancer samples were analyzed by western blot. **C** Quantification of IGSF9 protein expression. Relative intensity of IGSF9 in **B** was normalized to α-tubulin. Data are represented as the mean ± SD. The paired Student’s *t* test was used. ***P < 0.001. **D** Scores of IGSF9 immunohistochemistry staining in breast cancer specimens. Representative immunohistochemistry (IHC) staining intensity of IGSF9 in tumors and paired normal tissues. Scale bar, 20 μm. **E** Representative IHC staining intensity of IGSF9 in breast cancer and adjacent nontumorous tissue. Left scale bar, 200 μm, and right scale bar, 50 μm. **F**, **G** Quantification of IGSF9 IHC staining in specimens. The paired Student’s *t* test was used. ***P < 0.001. Low IGSF9 expression was associated with significantly worse prognosis of breast cancer patients. Relapse-free survival (RFS) (**H**) and overall survival (OS) (**I**) comparing low-expression group with high-expression of IGSF9 group were estimated using the Kaplan-Meier method and compared by the log-rank test. *n* = 107.

IGSF9 mRNA expression in 28 pairs of breast cancer tumors and adjacent normal tissues were detected by qRT-PCR. IGSF9 expression was markedly decreased in 75% (21/28) of breast cancer tumors, comparing with the corresponding adjacent normal tissues (Fig. 2A). Consistently, western blot analysis also showed a significant down-regulation of IGSF9 in breast cancer tumors (*P* < 0.001; Fig. 2B, C). Furthermore, we collected 160 pairs of breast cancer specimens with matched adjacent normal tissues from patients underwent curative resection for immunostaining. The distribution of IGSF9 is dispersed in the cells (Fig. 2D, E). The typical immunostaining images showed a significant downregulation of IGSF9 in breast cancer tumor compared with adjacent normal mammary epithelial cells (Fig. 2D–G).

To assess the clinical significance of IGSF9 expression in breast cancer, the expression of IGSF9 was scored. Patients were divided into IGSF9 high and low expression group according to the median protein level of IGSF9. Notably, low IGSF9 expression was significantly correlated with lymph node metastasis (*P* = 0.023) and advanced TNM (tumor-node-metastasis) staging (*P* = 0.030), but not age, tumor size or tumor differentiation (Table 1). In GSE16446 database, we found that low IGSF9 expression in breast cancer patients was associated with worse relapse free survival (RFS) (*P* = 0.037) and overall survival (OS) (*P* = 0.019), compared with those of high IGSF9 expression (Fig. 2H, I). Taken together, these data indicated that decreased IGSF9 expression correlated with poor prognosis and metastasis and could be a valuable indicator for poor prognosis in breast cancer patients.

**Table 1:** Correlation between IGSF9 expression and clinicopathologic characteristics of breast cancer patients.

Clinicopathologic characteristics		IGSF9 expression in tumor tissues		Total	*P* value
		Low expression	High expression		
		*n* (%)	*n* (%)		
Age	<50	42 (53.85)	36 (46.15)	78	0.427
	≥50	39 (47.56)	43 (52.44)	82	
Size	≤2 cm	30 (46.15)	35 (53.85)	65	0.349
	>2 cm	51 (53.68)	44 (46.32)	95	
Lymphatic metastasis^a^	−	38 (43.18)	50 (56.82)	88	**0.023**
	+	43 (61.43)	27 (38.57)	70	
Tumor differentiation	Low/medium	46 (46.46)	53 (53.54)	99	0.180
	High	35 (57.38)	26 (42.62)	61	
TNM stage	I	19 (39.58)	29 (60.42)	48	**0.030**
	II	31 (47.69)	34 (52.31)	65	
	III	31 (65.96)	16 (34.04)	47	
Total		81 (50.63)	79 (49.37)	160	

Pearson’s χ^2^ tests for all analyses. Bold items were considered statistically significant.

*IGSF9* immunoglobulin superfamily member 9, *TNM* tumor-node-metastasis.

^a^Some values were absent in TMA.

### *IGSF9* copy number and mutations do not alter *IGSF9* mRNA levels

Genetic alteration and epigenetic inactivation are two major causes for the loss of suppressor tumor genes. To explore genomic alteration of *IGSF9* in breast cancer, we analyzed the sequences in 9555 breast cancer cases from cBioportal database (2012 to 2021). The frequency of *IGSF9* copy number alteration was 0–21.26%, which were all amplification (Fig. S3A). Besides, only 5/16 studies from cBioportal database indicated *IGSF9* mutation may occur, and the frequency of *IGSF9* mutation was <3% in these datasets (Fig. S3B). No significant decrease of *IGSF9* mRNA with the shallow deletion of *IGSF9* was found (Fig. S3C). These data indicate that gene copy number is not a major factor for reduced *IGSF9* expression level in breast cancer. The location of the different mutations within *IGSF9* was shown in Fig. S3D, four mutations of which were predicated to be deleterious with the polymorphism phenotyping tool (Table 2). The analysis of COSMIC showed that no mutation at *IGSF9* promoter region containing the p53 binding site (Fig. S3E). mRNA expression of *IGSF9* mutations was uniformly distributed in three indicated datasets, indicating that *IGSF9* mutations did not affect its mRNA level in breast cancer (Fig. S3F).

**Table 2:** IGSF9 mutations in breast cancer carcinomas.

Study	Sample ID	Cancer Type	Protein Change	Functional Impact	Mutation Type	Copy#	Mut in Sample#
INSERM, PLoS Med 2016	MBC-29	Invasive Breast Carcinoma	R198Q	Deleterious	Missense	Gain	345
TCGA, PanCancer Atlas	TCGA-A2-A3Y0-01	Breast Invasive Ductal Carcinoma	R434P	Deleterious	Missense	Gain	147
TCGA, PanCancer Atlas	TCGA-AC-A23H-01	Breast Invasive Ductal Carcinoma	K651N	Tolerated	Missense	Gain	4260
TCGA, PanCancer Atlas	TCGA-AR-A2LE-01	Breast Invasive Lobular Carcinoma	D102Y	Deleterious	Missense	Gain	256
TCGA, PanCancer Atlas	TCGA-D8-A1XK-01	Breast Invasive Ductal Carcinoma	P351Hfs*30	Unknown	FS del	Gain	969
TCGA, PanCancer Atlas	TCGA-AN-A046-01	Breast Invasive Ductal Carcinoma	E934D	Medium	Missense	ShallowDel	5392
TCGA, PanCancer Atlas	TCGA-AN-A046-02	Breast Invasive Ductal Carcinoma	E422*	Unknown	Nonsense	ShallowDel	5392
TCGA, PanCancer Atlas	TCGA-A8-A09Q-01	Breast Invasive Ductal Carcinoma	L289P	Tolerated	Missense	Gain	129
TCGA, PanCancer Atlas	TCGA-A2-A0T5-01	Breast Invasive Ductal Carcinoma	T291P	Deleterious	Missense	Gain	1076
INSERM, PLoS Med 2016	MBC-137	Invasive Breast Carcinoma	W941*	Unknown	Nonsense	Diploid	41
INSERM, PLoS Med 2016	MBC-180	Invasive Breast Carcinoma	Q263*	Unknown	Nonsense	Amp	63
INSERM, PLoS Med 2016	MBC-39	Invasive Breast Carcinoma	V228F	Tolerated	Missense	Gain	94
INSERM, PLoS Med 2016	MBC-189	Invasive Breast Carcinoma	K1122N	Medium	Missense	Gain	935
TCGA, PanCancer Atlas	TCGA-EW-A2FV-01	Breast invasive Carcinoma (NOS)	P953Qfs*34	Unknown	FS del	Gain	3877
TCGA, PanCancer Atlas	TCGA-EW-A2FV-01	Breast invasive Carcinoma (NOS)	D644lfs*109	Unknown	FS del	Gain	3877
TCGA, PanCancer Atlas	TCGA-EW-A2FV-01	Breast invasive Carcinoma (NOS)	S200Afs*23	Unknown	FS del	Gain	3877

*Represents the termination codon.

### Proteasome-mediated degradation might contribute to decreased IGSF9 expression in breast cancer cells

To investigate the role of miRNAs in the regulation of IGSF9 expression at a post-transcriptional stage, two potential miRNAs targeting the 3′-UTR of *IGSF9* mRNA with high probability, miR-2355-5p and miR-8485, were identified from Target scan, miRDB and miRTarBase (Fig. S4A–C). However, neither hsa-miR-8485 nor hsa-miR-2355-5p inhibited luciferase activity of *IGSF9* 3′-UTR, suggesting that these two miRNAs were not involved in *IGSF9* regulation in breast cancer.

We next tested the stability of IGSF9 protein in breast cancer. CHX chase experiments showed that IGSF9 protein became less stable in the breast cancer cell than that in normal mammary cell MCF-10A (Fig. S4D). MG132 (a proteasome inhibitor), but not NH_4_Cl (a lysosome inhibitor) substantially increased IGSF9 protein levels in MCF-10A and MCF-7 cells (Fig. S4E, F). These data suggested that proteasome-mediated degradation might contribute to decreased IGSF9 protein level in breast cancer cells.

### Loss of IGSF9 promotes breast cancer metastasis

**Fig. 3:**
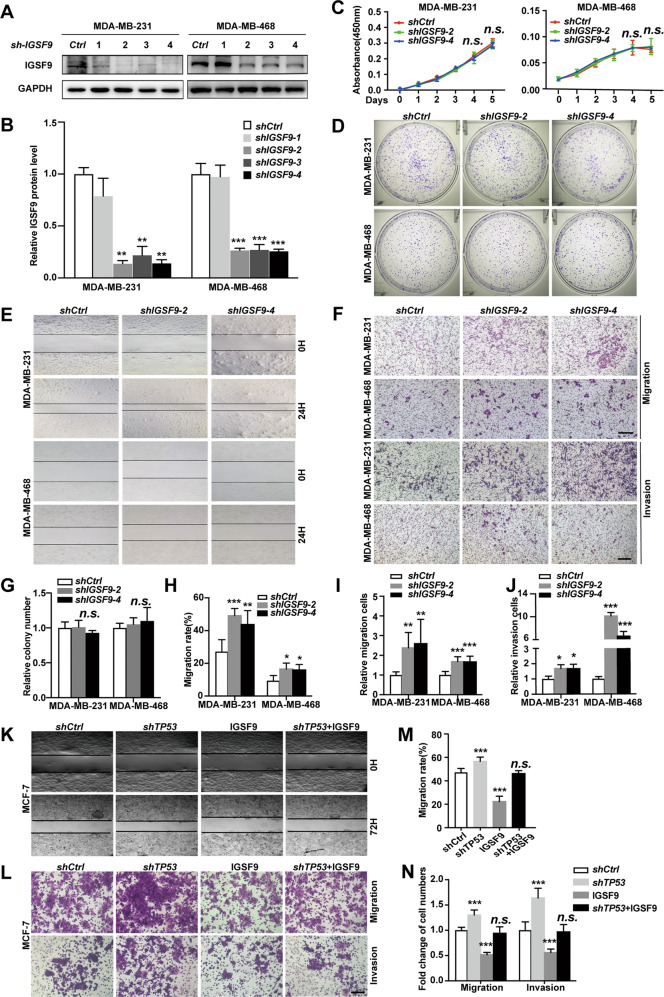
Loss of IGSF9 promotes breast cancer metastasis, but not proliferation. **A** *IGSF9* was knocked down in MDA-MB-231 and MDA-MB-468 cells. *shCtrl* and four *shIGSF9* lentiviral particles were used to generate MDA-MB-231 *shCtrl*, MDA-MB-231 *shIGSF9*-1/2/3/4, MDA-MB-468 *shCtrl*, and MDA-MB-468 *shIGSF9*-1/2/3/4 stable cell lines. IGSF9 protein was determined by western blot. **B** Quantification of the IGSF9 expression in mentioned stable cell lines. Data were represented as the mean ± SD. The Student’s *t* test was used; **P < 0.01, ***P < 0.001. **C** IGSF9 didn’t affect breast cancer cell proliferation. CCK-8 assays were performed with the stable cells above. Data were represented as the mean ± SD. The Student’s *t* test was used; *n.s*. indicated no statistically significance. **D**, **G** IGSF9 didn’t affect breast cancer cell colony formation. Relative colony numbers were counted and shown in **G**. Data were represented as the mean ± SD. The Student’s *t* test was used; *n.s*. indicated no statistically significance. *IGSF9* knockdown promoted the migration of breast cancer cells. Wound-healing assays were conducted with referred breast cancer cells (**E**). Quantification of **E** was performed by ImageJ and shown in **H**. Error bars denoted mean ± SD. The Student’s *t* test was used; *P < 0.05, **P < 0.01, ***P < 0.001. **F**, **I**, **J** *IGSF9* knockdown promoted the migration and invasion of breast cancer cells. Transwell assays were used to determine the migration and invasion abilities of these stable cell lines. Scale bar, 100 μm. Quantification of **F** was performed by ImageJ and shown in **I**–**J**, respectively. Error bars denoted mean ± SD. The Student’s *t* test was used; *P < 0.05, **P < 0.01, ***P < 0.001. IGSF9 overexpression rescued *TP53* knockdown induced migration (**K**–**N**) and invasion (**L**, **N**) of the referred stable cells. Scale bar, 100 μm. The Student’s *t* test was used; *P < 0.05, **P < 0.01, ***P < 0.001.

To investigate the role of IGSF9 in proliferation and metastasis of breast cancer cells, we stably over-expressed IGSF9 in MCF-7 and T47D cell lines, and knocked down *IGSF9* in MDA-MB-231 and MDA-MB-468 cell lines. IGSF9 protein levels were verified using western blot (Fig. S5A-B and Fig. 3A, B). Both CCK-8 proliferation and colony formation assays indicated that proliferation ability was not altered in IGSF9 over-expression/knockdown cells (Fig. S5C, D and Fig. 5C, D, G). However, over-expression of IGSF9 inhibited the migration of MCF-7 and T47D cells, while *IGSF9* knockdown promoted the migration of MDA-MB-231 and MDA-MB-468 cells, as measured by wound-healing and transwell migration assays (Fig. S5E, F and Fig. 3E, F, H, I). Consistently, transwell invasion assays indicated that over-expression of IGSF9 led to a significant decrease of cell invasion ability, while IGSF9 knockdown resulted in an increase of cell invasion in breast cancer cells (Figs. S5G and 3F, J). Together, these data indicated that loss of IGSF9 promoted breast cancer metastasis in vitro. Furthermore, over-expression of IGSF9 dampened *TP53*-knockdown induced MCF-7 cell migration and invasion (Fig. 3K–N), suggesting that IGSF9 could rescue breast cancer metastasis induced by *TP53*-knockdown.

### IGSF9 inhibits epithelial–mesenchymal transition (EMT) process in breast cancer cells

**Fig. 4:**
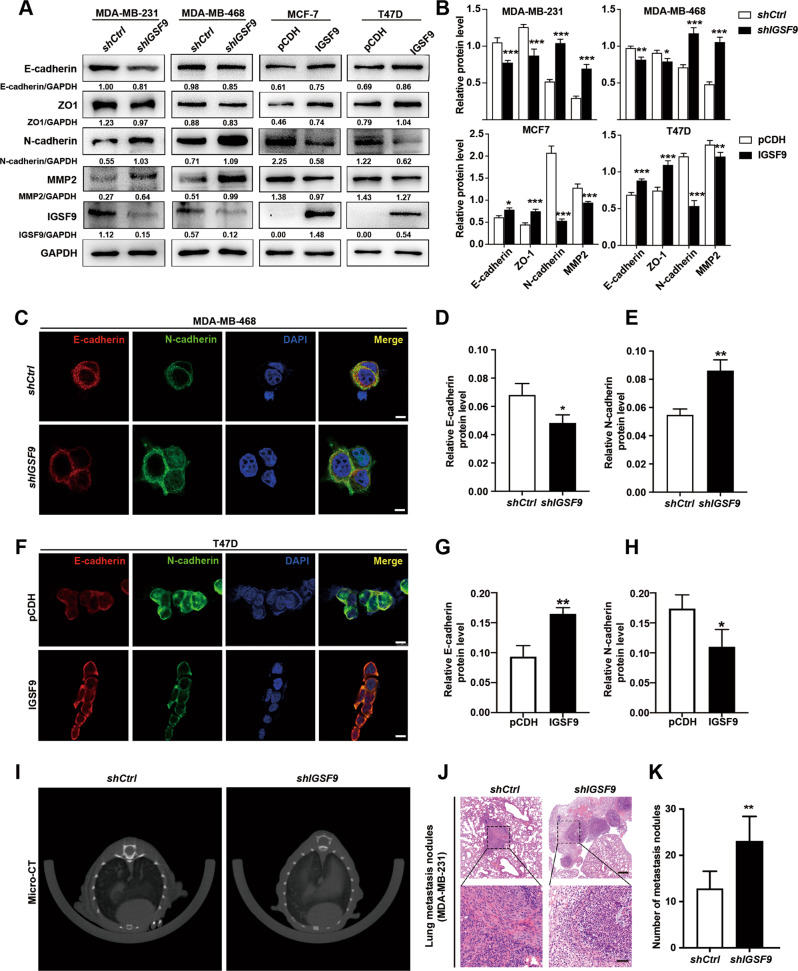
IGSF9 inhibits epithelial-mesenchymal transition (EMT) process in breast cancer. *IGSF9* knockdown promoted EMT in breast cancer cells. EMT markers in MDA-MB-231 and MDA-MB-468 stable cells were determined by western blot (**A**) and relative protein levels were normalized to GAPDH (**B**). Error bars denoted mean ± SD. The Student’s *t* test was used. *P < 0.05, **P < 0.01, ***P < 0.001. IGSF9 manipulation changes the protein expression of E-cadherin and N-cadherin in breast cancer cells. The mentioned stable cells were subjected to immunofluorescence (**C**, **F**). Red: E-cadherin, Green: N-cadherin, Blue: DAPI. Scale bars, 20 μm. Quantification analysis of E-cadherin and N-cadherin foci were performed by Image J (**D**, **E**, **G**, **H**). Error bars denote mean ± SD. The Student’s *t* test was used. *P < 0.05, **P < 0.01. **I** MDA-MB-231 cells were injected into tail vein of NOD-SCID mice to develop the lung metastasis model. At 4 weeks after injection, metastasis tumors were visualized by Micro-CT. **J** *IGSF9* reduction promoted lung metastasis of breast cancer cell in vivo. Hematoxylin-eosin (HE) staining images of lung metastatic nodules from each group were showed. Upper scale bar, 200 μm; lower scale bar, 50 μm. **K** Relative quantification of lung metastasis nodules from *shCtrl* and *shIGSF9* group. Values were mean ± SD. The Student’s *t* test was used; *n* = 5, *P < 0.05, **P < 0.01.

EMT plays critical and intricate roles in promoting tumor invasion and metastasis in epithelium-derived carcinomas, such as breast cancer, colorectal cancer. The typical EMT phenotypes, down-regulation of E-cadherin and ZO-1, and up-regulation of N-cadherin and MMP2, were found as well in *IGSF9*-knowdown breast cancer cells with western blot analysis (Fig. 4A, B) and immunofluorescence assay (Fig. 4C–E). In contrary, EMT phenotypes were inhibited in IGSF9-overexpressed breast cancer cells (Fig. 4A, B, F–H). We next generated lung metastasis model via tail vein injection of breast cancer stable cell suspensions. MDA-MB-231 cells with *IGSF9*-knockdown led to an increase in the lung metastasis burden evidenced by both Micro-CT and hematoxylin-eosin (HE) staining for lung metastases nodes (Fig. 4I–K).

### IGSF9 inhibits FAK/AKT signaling via its interaction with FERM domain of FAK

**Fig. 5:**
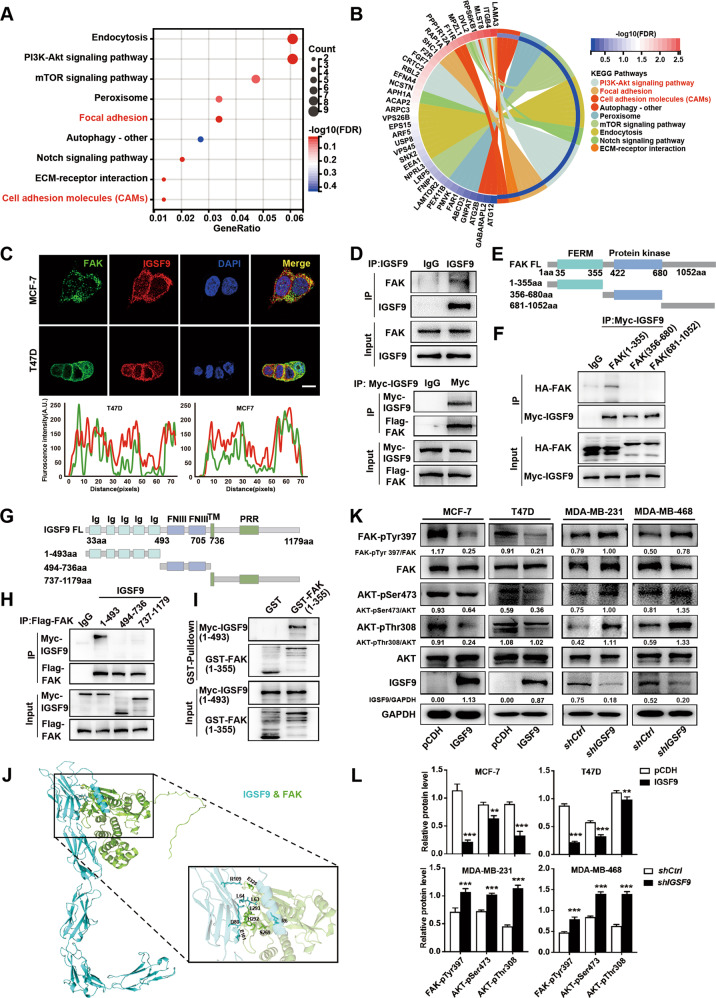
IGSF9 inhibits FAK/AKT signaling via its interaction with FERM domain of FAK. **A** KEGG pathways are enriched by differentially expressed genes (DEGs) regulated by IGSF9. Dot size represented the ratio of number of differential genes to the total number of corresponding pathways genes. **B** The KEGG pathway of DEGs enriched the chord diagram. **C** Immunofluorescence exhibited co-localization of IGSF9 and FAK. Scale bar: 25 μm. A line profile plot showed the pixel intensities of FAK and IGSF9 along a dashed line presented in the merged figure by Image J. **D** IGSF9 interacted with FAK. Co-immunoprecipitation of endogenous IGSF9 with FAK was performed in MCF-7 cells (upper panel). Myc-IGSF9 and Flag-FAK-expressing plasmids were transfected into HEK293T cells. Co-immunoprecipitation was conducted by anti-Myc (bottom panel). 1-355 aa fragment of FAK interacted with IGSF9. HEK-293T cells were transfected with Myc-IGSF9 and HA-tagged FAK truncated plasmids (**E**). Cell lysates were subject to co-immunoprecipitation and western blot (**F**). 1-493 aa fragment of IGSF9 interacted with FAK. HEK-293T cells were transfected with Flag-FAK and Myc-tagged IGSF9 truncated plasmids (**G**). Cell lysates were subject to co-immunoprecipitation and western blot (**H**). **I** The direct interaction between 1-493 aa fragment of IGSF9 and 1-355 aa fragment of FAK was validated by GST pull-down assay. **J** The 3D structures of IGSF9-FAK complex. IGSF9 over-expression inhibits FAK/AKT signaling activity, while *IGSF9* knockdown activated FAK/AKT signal. The indicated signal molecules in stable cells were determined by western blot (**K**). Quantification of protein level was performed by Image J (**L**). Error bars denoted mean ± SD. The Student’s *t* test was used. **P < 0.01, ***P < 0.001.

To explore the molecular mechanisms underlying the tumor suppressive effect of IGSF9, we analyzed the differential gene expression between IGSF9 high and low expression samples in GSE27830. KEGG enrichment pathway analysis was conducted using DEGs. Enriched pathways mainly included focal adhesion, PI3K-AKT signaling, ECM-receptor interaction, and cell adhesion molecules (Fig. 5A, B).

FAK acts as both signaling kinase and cell adhesion-associated scaffold protein involved in breast cancer metastasis [27, 36]. The structure of IGSF9 renders it capable of serving as a potential cell-surface receptor and transducing outside signal into intracellular pathway. Immunofluorescence exhibited co-localization of IGSF9 and FAK (Fig. 5C). Moreover, co-immunoprecipitation assay showed the endogenous and exogenous interaction between IGSF9 and FAK in MCF-7 cells and HEK-293T cells, respectively (Fig. 5D). To identify the regions in FAK that interact with IGSF9, we expressed various HA-tagged FAK fragments together with IGSF9 (Fig. 5E), and performed co-immunoprecipitation experiments. As shown in Fig. 5F, FAK (1-355 aa) containing FERM domain can bind with IGSF9. Meanwhile, we also expressed various Myc-tagged IGSF9 fragments together with FAK (Fig. 5G). As shown in Fig. 5H, IGSF9 (1-493 aa) containing five Ig domains can bind with FAK. In vitro-translated IGSF9 (1-493 aa) was pulled down by purified GST-FAK (1-355 aa) fusion protein (Fig. 5I), indicating a direct interaction between IGSF9 (1-493 aa) and FAK (1-355 aa). We predicted IGSF9–FAK complex structural model based on the X-ray crystal structure from Protein Data Bank. Docking modeling data suggested that amino acids R6, L63, L64, D80, E101, and R109 of IGSF9, as well as amino acids S269, H292, L293, and E325 of FAK, are critical for the interaction (Fig. 5J). The activities of key molecules in FAK/AKT pathway were thus investigated in cells with IGSF9 over-expression or knockdown. We found that p-FAK (Y397), p-AKT (S473), and p-AKT (T308) were significantly decreased, indicating an inhibition of FAK/AKT signaling in IGSF9 over-expression MCF7 and T47D cells (Fig. 5K-L). Conversely, FAK/AKT signaling was activated in *IGSF9*-knockdown MDA-MB-231 and MDA-MB-468 cells (Fig. 5K-L). These results suggested that IGSF9 interacted with FAK and antagonized FAK/AKT signaling.

### PND1186, FAK inhibitor, inhibits breast cancer metastasis induced by *IGSF9* knockdown in vitro and in vivo

**Fig. 6:**
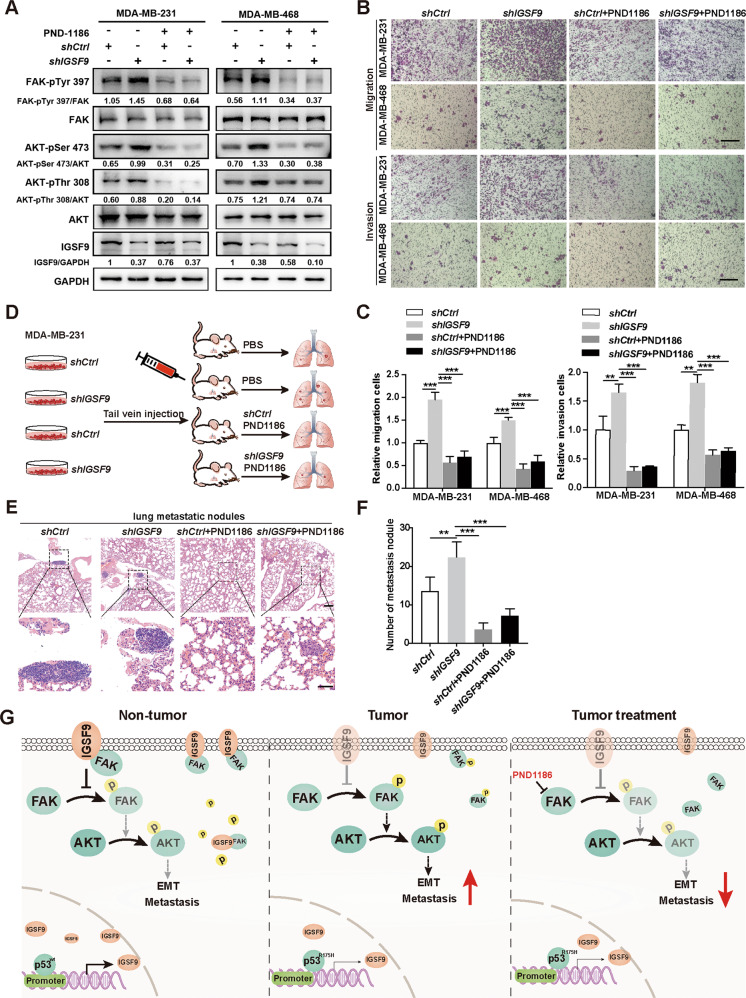
PND1186, FAK inhibitor, inhibits breast cancer metastasis induced by *IGSF9* knockdown in vitro and in vivo. **A** PND1186, FAK inhibitor, could reverse IGSF9 reduction induced activation of FAK/AKT signaling pathway in breast cancer cells. The phosphorylation of the indicated signal molecules in stable cells was determined by western blot. **B** PND1186 abolished the migration and invasion induced by *IGSF9* knockdown. Transwell assay were conducted in the mentioned breast cancer cells. Scale bars, 100 μm. **C** Quantification of migration and invasion (**B**) using Image J. Data were represented as the mean ± SD. The Student’s *t* test was used. **P < 0.01, ***P < 0.001. **D** Schematic depicting the establishment of a lung metastatic tumor model. BALB/c anthymic nude mice were injected breast cancer cells (MDA-MB-231 *shCtrl*/*shIGSF9*) through the tail vein; PND1186 (50 mg/kg) or PBS was administered by oral gavage two times a day for 4 weeks. PND1186 blocked the effect of IGSF9 knockdown promoted lung metastasis of breast cancers. Representative HE staining images of lung tissues from each group were shown (**E**). Upper scale bar, 200 μm; lower scale bar, 50 μm. Quantification of metastatic nodules (**F**). Error bars denote as mean ± SD. The Student’s *t* test was used. **P < 0.01, ***P < 0.001. **G** A schematic model illustrated the role of IGSF9 in EMT process and metastasis of breast cancer. In the normal tissue, wild-type p53 acting as a transcriptional factor bound to the promoter region of *IGSF9* and promoted its transcription. Increased IGSF9 interacted with FAK to block its phosphorylation, leading to decreased downstream protein AKT phosphorylation (left panel). Consequently, EMT and metastasis were halted. In breast cancer, p53 with R175H mutation was deficient in promoting IGSF9 transcription. IGSF9 reduction promoted FAK phosphorylation and activation of AKT, thereby EMT process and metastatic ability in breast cancer (middle panel). When breast cancer patients with low IGSF9 expression were treated with PND1186, a specific FAK inhibitor, the reduced FAK activity then led to attenuated EMT and metastasis (right panel).

PND1186, a specific FAK inhibitor, was used to treat MDA-MB-231 and MDA-MB-468 cells. Western blot analysis indicated that IGSF9 loss-mediated FAK/AKT signaling activation, was remarkably blocked in the presence of PND1186 (Fig. 6A). Transwell migration and invasion assays also demonstrated that administration of PND1186 dramatically reversed *IGSF9* knockdown-induced tumor migration and invasion of MDA-MB-231 and MDA-MB-468 cells (Fig. 6B, C). Moreover, we developed a lung metastasis model by injecting MDA-MB-231 cells into the tail vein in 4-6 weeks female NOD-SCID mice (Fig. 6D). After 6 weeks, the mice were sacrificed and lung tissue sections were stained with HE (Fig. 6E). The numbers of metastatic lesions in lungs showed that *IGSF9* knockdown accelerated tumor metastasis, which could be reversed by PND1186 treatment (Fig. 6F). Together, these data indicate that IGSF9 loss induces metastasis of breast cancer via activating FAK/AKT signaling. PND1186 might apply for breast cancer treatment in the future.

## Discussion

In this study, we identified a novel p53 target gene *IGSF9*, which is frequently lost in breast cancer patients and is associated poor prognosis. Loss of IGSF9 promotes breast cancer metastasis in vitro and in vivo. In breast cancer cells, reduced wild-type p53 or mutated p53 (R175H) down-regulated IGSF9 protein expression. A decrease of IGSF9 releases FAK, activates FAK/AKT signaling and promotes EMT process, resulting in cancer metastasis (Fig. 6G). FAK inhibitor PND1186 could inhibit *IGSF9* knockdown-induced breast cancer metastasis in vitro and in vivo.

IGSF9 family proteins show limited expression in normal tissues. According to PaxDb, mammalian members of the IGSF9 family belong to the bottom 5% of expression level [37]. The role of IGSF9 in cancer appears to be tissue context-specific. IGSF9 shows low expression in liver hepatocellular carcinoma, skin cutaneous melanoma, testicular germ cell tumors, as well as in breast cancer which was reported in the present study. On the other hand, IGSF9 shows high expression in bladder urothelial carcinoma, ovarian serous cystadenocarcinoma, pancreatic adenocarcinoma, uterine corpus endometrial carcinoma [38]. However, the regulation of IGSF9 expression remains unclear. In this study, we demonstrated that p53 functions as a transcription factor of IGSF9 and regulates IGSF9 expression in breast cancer.

As a transcription factor, p53 regulates tumor-related genes expression, such as CDKN1A, a cyclin-dependent kinase inhibitor [39], and GAS7, a breast cancer metastasis suppression gene [9]. Alterations of *TP53* are the most common genetic changes found in breast cancer and play a central role in cancer process [40]. The vast majority of p53 mutant proteins comes from missense mutations mostly within the DNA-binding domain of *TP53* [41]. Here we found that wild-type p53, but not R175H mutation within the DNA binding domain of p53, can bind to IGSF9 promoter, activating the transcription of IGSF9. Compared with wild-type p53, mutant p53 has a prolonged half-life, whereas cannot recognize wild-type p53 DNA-binding sites [42, 43]. Models for mutant p53 regulated transcriptional activity include: i) mutant p53 binds the regulatory regions of its target genes through a specific and yet unknown consensus sequence for DNA binding; ii) mutant p53 interacts with a specific transcription factor that drives its target gene [41]. Chang et al. reported that wild-type p53 trans-regulated *GAS7* gene expression; while mutated p53 significantly reduced *GAS7* expression [9]. Mutant p53 might sequester and inactivate proteins involving in genomic instability and the resistance to anticancer treatments, leading to increased metastasis.

FAK is a cytoplasmic protein tyrosine kinase. Upon stimulation by integrins and a broad range of growth factors and chemokines, canonical FAK signaling is activated to facilitate the formation and turnover of focal adhesions. FAK is autophosphorylated at Y397 to create a binding site for some SH2-containing molecules, including Src, PI3K, Grb7, and PLCγ [44]. When FAK is associated with Src upon Y397 autophosphorylation, it can (i) lead to Src conformational change and activation, which subsequently phosphorylates other sites of FAK, including Y407, Y576, and Y577, to maximize FAK kinase activity [45]; (ii) provide binding sites for other signaling molecules. The interaction between the FERM and kinase domain can maintain FAK autoinhibition state. IGSF9 was shown to mediate dendrites outgrowth and spine formation by interaction with MRCKβ [46]. Here, we discovered the direct interaction between Ig domain of IGSF9 and FERM domain of FAK. Loss of IGSF9 enhances FAK kinase activity, activates AKT, leading to breast cancer metastasis. It seems that binding IGSF9 to FAK enhances the autoinhibition of FAK kinase activity. Increased FAK expression and activity are associated with breast cancer progression and poor prognosis. Interestingly, nuclear FAK recruits p53, increasing p53 polyubiquitylation (Ub) and degradation [47], thereby preventing p53 transcription activity. This event would further decrease IGSF9 expression and facilitate cancer metastasis.

Small molecule FAK inhibitors are emerging as promising chemotherapeutics. FAK inhibition in mouse models prevents tumor growth, metastasis, vascular permeability and angiogenesis [48]. We found that PND1186, a FAK inhibitor, could inhibit breast cancer metastasis induced by *IGSF9* knockdown in vitro and in vivo. In line with our work, Walsh et al. reported that PND1186 blocked FAK Tyr-397 phosphorylation in vivo and exhibited antitumor efficacy in orthotopic breast carcinoma mouse tumor models [36]. However, the scaffolding functions of FAK are not blocked by FAK inhibition, even possibly enhanced by it [47], thereby leading to unpredictable therapy outcomes. The kinase-independent functions of FAK must be taken into consideration when designing or testing approaches to inhibit FAK kinase activity for therapeutical applications.

Collectively, we identified a new p53 target, IGSF9, in promoting breast cancer metastasis through EMT process mediated via FAK/AKT signal. This study discovered a feasible prognostic biomarker IGSF9, and at the same time sheds light on the potential new strategies for developing treatment for breast cancer patients.

## Materials and methods

### Patient and specimens

Randomly chosen 28 pairs of freshly collected tumor tissues and adjacent normal tissues coming from breast cancer patients who underwent surgical resection and pathologically diagnosed with breast cancer in Zhongshan Hospital of Fudan University (Shanghai, China) were used for quantitative reverse transcription polymerase chain reaction (qRT-PCR) and western blot analysis. A total of 160 breast cancer patients tissue microarray and patients’ clinicopathological characteristics: age, tumor size, TNM staging *et* were obtained from Shanghai superbiotek technology company. RFS was defined as the time interval from surgery to recurrence, and overall survival (OS) as time interval from surgery to death. The study was approved by the Institutional Ethics Committee of Fudan University, and written informed consent was obtained from each patient before clinical data analysis.

### Plasmids and transfection

The IGSF9 plasmids have been previously described [46]. FAK sequence were cloned into GV492 vector, and mutant sequences were cloned into pcDNA3.1 for overexpression. pGL3 vector for wild-type and mutant *IGSF9* 3′-UTR, and pGEX-KG for GST pull-down assays. Deletion and site-directed mutations were incorporated into plasmid DNA using Q5 Site-Directed Mutagenesis kit (New England Biolabs, Massachusetts, USA). Primers used in this study are listed in Table S1.

HEK-293T, human breast cancer cell lines MCF-7, T47-D, MDA-MB-231, MDA-MB-468, MDA-MB-436, BT549, and mammary epithelial cell MCF-10A were all purchased from Chinese Academy of Sciences Cell Bank. MCF-7 and HEK-293T were maintained in Dulbecco’s Modified Eagle Medium (Invitrogen Corporation, CA, USA) supplemented with 10% of Fetal Bovine Serum (FBS; Invitrogen Corporation, CA, USA) and with 1% penicillin/streptomycin (P/S; Invitrogen Corporation, CA, USA) at 37 °C in a 5% CO_2_ atmosphere. MDA-MB-231, MDA-MB-468, MDA-MB-436 were all maintained in L-15 (Invitrogen Corporation, CA, USA) supplemented with 10% of FBS and with 1% P/S at 37 °C without CO_2_. MCF-10A were maintained in Ham’s F12 (Sigma-Aldrich, St. Louis. MO) supplemented with 5% of Horse serum, 1% P/S, 10 μg/mL insulin, 0.5 μg/mL dexamethasone, 100 ng/mL cholera toxin (CT), 20 ng/mL Epidermal Growth Factor at 37 °C in a 5% CO_2_ atmosphere. The MDA-MB-231, MDA-MB-468, MCF-7, and T47D were infected by the shRNA expressing lentivirus for IGSF9 and the IGSF9 over-expression lentivirus respectively, and stable clones were selected by puromycin (Sigma Aldrich, St. Louis. MO). Breast cancer cells and HEK-293T cells were transfected with the over-expression plasmids and siRNA using Lipofectamine 3000 (Invitrogen Corporation, CA, USA).

### RT-qPCR

Total RNA from cells and breast tissues was extracted with TRIzol^TM^ (Thermo Fisher Scientific). cDNA was retro-transcribed using Primescript^TM^ RT Master Mix (TaKaRa, Japan). Quantitative PCR (qPCR) reactions for IGSF9 and GAPDH were performed with an ABI Prism 7500 instrument (Applied Biosystem, USA), and amplified in SYBR Green Mix (TaKaRa, Japan) with the corresponding primers. The primers were listed in Table S1. The relative gene expressions were determined by 2^-△△Ct^ method [49]. Experiments were performed in triplicate, and results were presented as mean value ± SD.

### Luciferase reporter assays

The promoter sequence of *IGSF9* gene (from −3000 to +50 bp) was obtained from the http://genome.ucsc.edu. The sequences of the promoter were amplified by PCR, and the fragments were cloned into the pGL3-basic vector. Cells were co-transfected with pGL3-basic or pGL3 IGSF9-Luc together with pRL-TK plasmid. Cell samples were harvested at 48 h post-transfection, lyzed with lysis buffer and measured luciferase activity using the luciferase detection kits (Promega, USA) according to the manufacturer’s instructions. Relative luciferase activity was detected using a Lumat LB 9507 luminometer (Berthold Technologies, Germany). All experiments were performed in three independent replicates.

### Chromatin Immunoprecipitation (ChIP) assays

ChIP experiments were performed using the ChIP assay kit (17-295, Merck Millipore, Darmstadt, Germany) according to the manufacturer’s instructions. Breast cancer cells were seeded at a density 1 × 10^7^ cells in 100 mm cell culture dish, and grown overnight to 95% confluence. Cells treated for 10 min at room temperature were crosslinked by adding formaldehyde into cell culture medium to a final concentration of 1%, and washed with cold 1 × PBS buffer containing protease inhibitors. Then the cells were sonicated to shear DNA for 30 min on ice, and immunoprecipitated with anti-p53 antibody and control IgG overnight at 4 °C. DNA fragments were extracted and purified from the immunoprecipitates by the instructions and analyzed by RT-PCR using the primers designed to recognize and amplify the IGSF9 promoter. See Table S2 for primer information.

### Western blotting

Total protein was extracted from cells or tissues were lysed in RIPA buffer (Beyotime Biotechnology, Shanghai, China) with 1 × protease inhibitor cocktail (Roche), incubated for 30 min on ice, and centrifuged 10 min at 12,000 rpm at 4 °C. Protein concentrations in supernatants were detected using a BCA Protein Assay Kit (Vazyme Biotech, Nanjing, China). 10 μg of protein lysates were run on 8–10% sodium dodecyl sulfate-polyacrylamide gel electrophoresis (SDS-PAGE), and transferred to 0.45 μm PVDF membranes (Millipore, Darmstadt, Germany). The PVDF membranes were incubated in 5% (w/v) non-fat dry milk resuspended in 0.1% TBST for 1.5 h at room temperature, and the membranes were incubated with primary antibodies overnight at 4 °C. After washing primary antibodies with TBST, PVDF membranes were incubated in horseradish peroxidase (HRP)-conjugated secondary antibodies for 1 h at room temperature. The primary and secondary antibodies, and their dilutions were listed in Table S2. After washing the membranes four times with TBST, bands were visualized using ECL Western Blotting Detection Reagents (Tanon, Shanghai, China).

### Immunohistochemical (IHC) staining and scoring

The paraffin-embedded specimens were sliced into 4 μm thick, and mounted on poly-L-lysine-coated glass slides. Then sections were deparaffinized, rehydrated with xylene and alcohols. Antigen retrieval was performed by placing sections in EDTA solution (pH = 9.0) and heating at 100 °C for 20 min, followed by cooling at room temperature for 30 min. Placing the sections in 0.03% H_2_O_2_ at 37 °C for 15 min were used to block endogenous peroxidase activity. After blocking in TBS with 5% bovine serum albumin (BSA) at room temperature for 30 min, the sections were incubated with the anti-IGSF9 antibody overnight at 4 °C, and then incubated HRP-conjugated secondary antibodies. The antibodies and dilutions used were listed in Table S2. Immunoreactivity was quantified using the staining intensity (0, negative; 1, weak; 2, moderate; 3, strong) and the percentage of positive stained tumor cells (0, <5%; 1, 6–25%; 2, 26–50%; 3, 50–75%; 4, 76–100%). The scores were obtained by multiplying the staining intensity and the percentage of positive stained cells [50].

### Immunofluorescence

The breast cancer cells were seeded on coverslips. After 24 h, cells were fixed with 4% paraformaldehyde at 4 °C for 30 min, and permeabilized/nonpermeabilized with 0.3% triton X-100 for 10 min. After blocking in TBS with 5% BSA for 1 h at room temperature, cells were incubated with primary antibodies overnight at 4 °C, then incubated with secondary antibodies at room temperature for 2 h. DAPI was used to dye cell nuclei. The antibodies and dilutions used were listed in Table S2. Images were taken with Confocal microscope.

### Co-immunoprecipitation and GST pull-down assay

Cells were cultured in 100 mm dishes, harvested at 90% confluence. After washing three times with cold PBS, cells were lysed in IP lysis buffer with 1×protease inhibitor cocktail, and incubated for 90 min on ice, then centrifuged 10 min at 12,000 rpm at 4 °C. For co-immunoprecipitation assays, cells lysates were immunoprecipitated with the anti-myc mouse monoclonal antibody and normal IgG followed by adsorption to protein A/G beads (Santa Cruz, Texas, USA), incubating overnight at 4 °C on a rotating rocker. After washing three times with cold PBS, the samples were analyzed by western blotting and 5% of input samples were used as a positive control. GST pull down assay was performed as described previously [46]. GST protein and GST-FAK (1-355) fusion protein were expressed in bacterial BL21 cells. Cells were lysed, and GST fusion proteins were captured by Glutathione-Sepharose 4B beads at 4 °C for 1 h. To investigate the interaction between IGSF9 and FAK (1–355), equal amounts of GST or GST- FAK (1-355) fusion protein beads were incubated with in vitro translated IGSF9. After washing with ice-cold wash buffer, the proteins were eluted from the beads and detected by western blot.

### Cell proliferation and colony formation assay

Cells (1 × 10^3^) were seeded into 96-well plate. CCK-8 solution was added in each well and incubated for 1 h at 37 °C. The absorbance at 450 nm was measured using a fully automated microplate reader (ELx800TM, BIO-TEK Instruments, Minneapolis, MN, USA). Cells (1 × 10^3^) were seeded into six-well plate, and were grown 2 weeks for detecting colony formation ability. The cells were fixed by 4% paraformaldehyde and stained with crystal violet (Beyotime Biotechnology, Shanghai, China).

### Wound-healing, transwell migrations, and invasion assays

For wound-healing assay, breast cancer cells were seeded in a 6-well plate and cultured to reach 85% confluent. Then cells were scraped by a sterile tip of a 10 μl pipette to generate an artificial wound. Photographed and measured the wound at 0 h, 24 h, 48 h, 72 h, and 96 h, respectively. The cell migration rates were obtained by calculating the wounds width changing. For the transwell migration assay, 2 × 10^4^ cells were resuspended in 200 μl serum-free medium and added to the upper compartments of transwell chambers (8 μm pore filter, Corning, NY, USA). Putting the transwell chamber in 24-well plate, and the lower compartments were filled with 20% serum medium (600 μl/well). After incubation at 37 °C for 12 h or 24 h, the migrated cells had passed through the pore to the lower membrane, and the transwell chamber lower membranes were fixed and stained with 0.1% crystal violet, photographed using an IX71 inverted microscope (Olympus Corp, Tokyo, Japan). For the transwell invasion assay, the upper compartments of the transwell chamber were coated by 30 μg matrigel (BD Biosciences, Franklin Lakes, NJ), the subsequent procedures were performed as the transwell migration assay. All experiments above were performed in three independent triplicates.

### Animals and lung metastatic tumor model

The pre-specified minimum sample size was calculated according to the approval of Shanghai Medical Experimental Animal Care Commission. Animals were excluded from the analysis if they died from other reasons than tumor metastasis (for example, sickness). The possibility of these events was considered over the sample size estimation. BALB/c athymic nude mice (female, 4–6 weeks old and specific pathogen free) were purchased from Shanghai SLAC laboratory Animal Co., Ltd (Shanghai, China), and were maintained in a pathogen-free animal facility under standard conditions. All animal procedures were performed according to the criteria outlined in the “Guide for the Care and Use of Laboratory Animals” prepared by the National Academy of Sciences and published by the National Institutes of Health (NIH publication 86-23 revised 1985). Studies were approved by the Shanghai Medical Experimental Animal Care Commission.

To establish experimental lung metastasis model in BALB/c athymic nude mice, breast cancer cells (1 × 10^6^ in 150 μL PBS) from different treated groups were injected through the tail vein. The mice were randomly allocated to four groups according to the breast cancer cells received: MDA-MB-231-*shCtrl*, MDA-MB-231-*shIGSF9*, MDA-MB-231-*shCtrl* + PND1186, MDA-MB-231-*shIGSF9* + PND1186. For each group, six mice were recruited. For PND1186 group, 50 mg/kg PND1186 was administered by oral gavage two times a day. Then mice were sacrificed after 6 weeks and whole lung of each mouse was removed, fixed with 4% paraformaldehyde, embedded in paraffin, and serially sectioned. Paraffin sections were stained with HE, randomly selected fields images were taken using a spectral imaging system (Vectra Polaris; PerkinElmer) and the number of metastatic lesions in lungs analyzed using ImageJ software by three performing scientist blinded to the knowledge of the experimental methods.

### Screening results of potential target genes

The potential target genes of *TP53* were predicted by Chip-Atlas (http://chip-atlas.org/), GTRD (http://gtrd.biouml.org/), hTFtarget (http://bioinfo.life.hust.edu.cn/ hTFtarget/) and Harmonizome (https://maayanlab.cloud/Harmonizome/). The overlapping potential genes from the mentioned databases were obtained using the Draw Veen Diagram (http://www.ehbio.com/ImageGP/) online tool. Gene Ontology (GO) and Kyoto Encyclopedia of Genes and Genomes (KEGG) enrichment analyses were conducted in Metascape website (http://metascape.org/gp/index.html#/main/step1). To further capture the relationships between the terms, a series of enriched terms have been chosen and rendered as a network plot, where terms with a similarity >0.3 are connected by edges. The terms with the best *P* values from each of the 20 cluster were selected.

### GEO series

The gene expression profiles of GEO series 27830 (GSE27830) were downloaded from the GEO database (https://www.ncbi.nlm.nih.gov/geo/query/acc.cgi) using the GEO query package, and the probes corresponding to multiple molecules were deleted. When the probes corresponding to same molecules were encountered, probes with the largest signal values were reserved. The gene names were annotated to gene probes of the platform by referring to the GPL570 platform.

### KEGG pathway analysis for DEGs

Differentially expressed genes (DEGs) were detected between IGSF9 high and low expression breast cancer group from GSE27830 dataset using package DESeq2. The adjusted *P* value < 0.05 and |log2FC| > 0.5 as the threshold. Genes positively related with IGSF9 were used to analyze the functional enrichment of IGSF9-interacted genes. Kyoto Encyclopedia of Genes and genomes (KEGG) analysis was conducted with R package “cluster Profiler”.

### The structural analysis of IGSF9/FAK complex

The structural models of IGSF9 (1-493 aa; Template ID: Q05BQ1) and FAK (1-355 aa; Template ID: Q05397) were modeled with I-TASSER, FR-t5-M, and FALCON software. The IGSF9-FAK docking candidate interfaces were performed using ISPRED4. According to the candidate interfaces, IGSF9-FAK complex were docked with ZDOCK v3.0.2f. We selected highest-confidence complex model from top 10 candidate models using ZDOCK and SPR. All structure models were exhibited by PyMol Software (www.pymol.org).

### Statistical analysis

All statistical analyses were carried out using SPSS version 22.0. Pearson’s Chi-square test was performed to analyze the correlations between IGSF9 expression and clinicopathological characteristics. OS and TTR curves were depicted using Kaplan-Meier analyses (log-rank test). Differences between groups were compared by using a two-tailed Student’s *t* test. All experiments were performed in triplicate, and the data were presented as mean value ± SD. A value of *P* < 0.05 was considered statistically significant.

## Supplementary information

Supplemental material
