# Supplementary material for: Immunoglobulin superfamily 9 (IGSF9) is trans-activated by p53, inhibits breast cancer metastasis via FAK
Source: Oncogene. 2022 Sep 10;41(41):4658–72. doi: 10.1038/s41388-022-02459-8 (PMC9546770; doi:10.1038/s41388-022-02459-8)
Supplement: Supplementary file 1 — Supplemental material [file 41388_2022_2459_MOESM1_ESM.docx]

**Immunoglobulin superfamily 9 (IGSF9) is trans-activated by p53, inhibits breast cancer metastasis via FAK**

Yaohua Li^1,2*^, Yiran Deng^1*^, Yannan Zhao^1,3^, Si Zhang^1^, Li Zhang^1^, Biyun Wang^3^, Yingying Xu^1#^, She Chen^1#^

Table of Contents

Supplementary Fig. 12

Supplementary Fig. 23

Supplementary Fig. 34-5

Supplementary Fig. 46

Supplementary Fig. 57

Supplementary Table S18-10

Supplementary Table S211


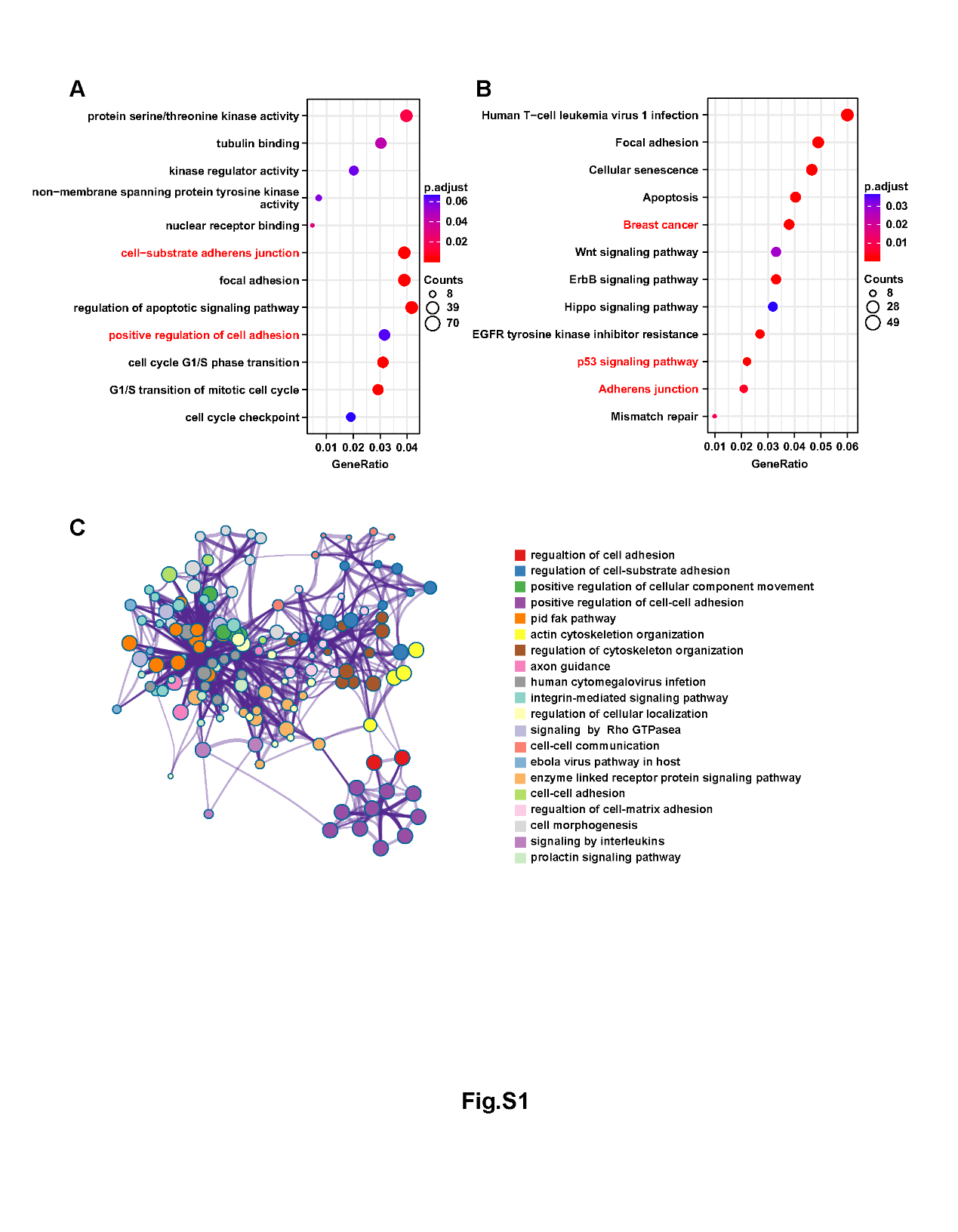


Figure S1. **Function enrichment analysis of p53 target genes**

**A** GO enrichment analysis predicted the function of p53 target genes in three domains: BP, CC, and MF, with adjust *P* **<**0.05 considered significant. **B** KEGG pathway enrichment bubble plot. Dot size represents the ratio of number of p53 target genes to the total number of corresponding pathways genes. **C** An interaction network of the top 20 enrichment terms. It is colored by cluster-ID. Distinct colors are various enrichment pathways of p53 target genes.


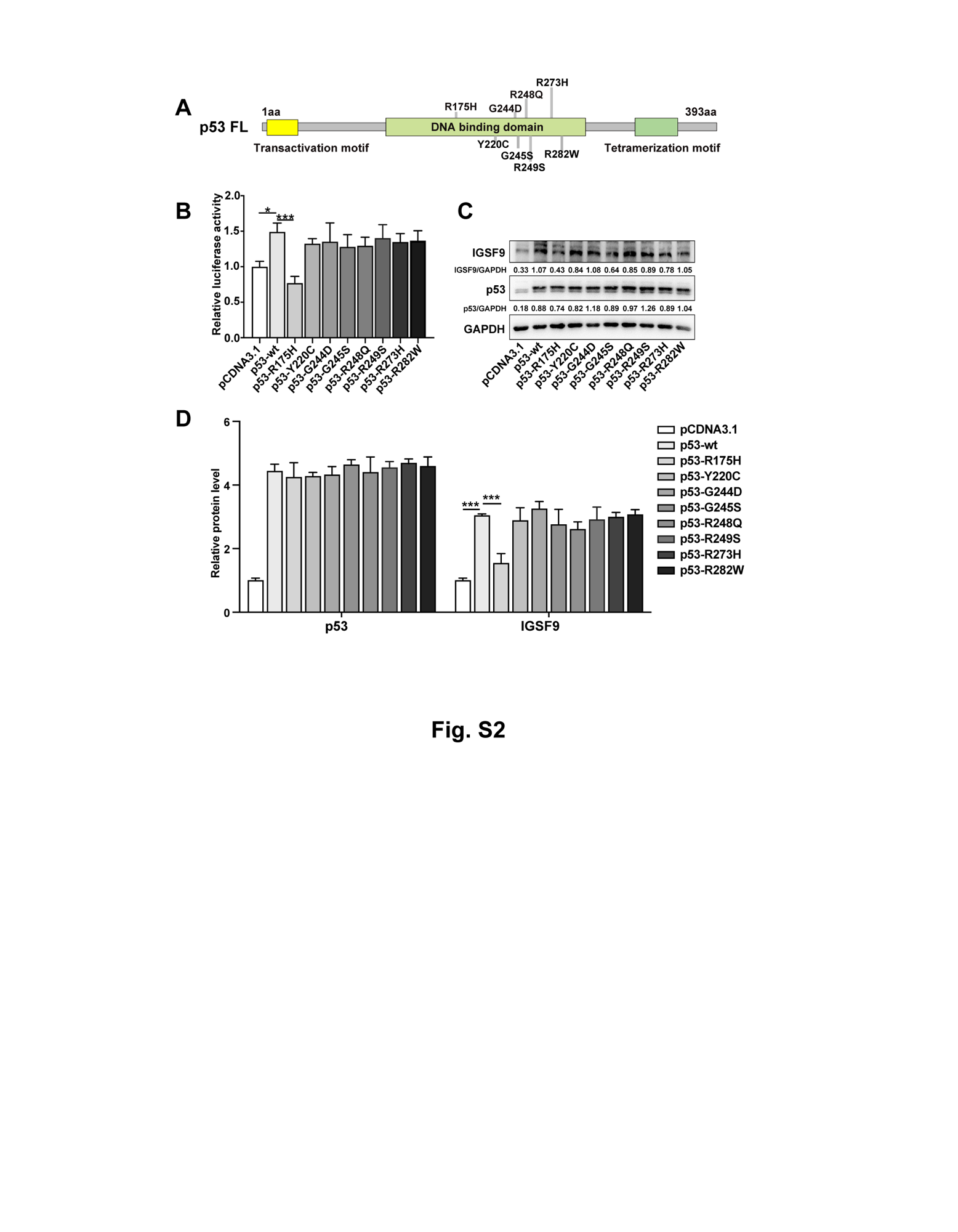


**Figure S2. p53 R175H decrease the expression of IGSF9 compared with wild-type p53**

**A** Schematic diagram illustrating the protein domains of p53, and eight mutation hotspots. Luciferase assay (**B**) and western blot (**C**) were conducted with MCF-7 cell. Quantification **(D)** was performed by ImageJ. Error bars denote as mean ± SD. The Student’s *t*-test was used; **P* **<**0.05, ****P* **<**0.001.

**
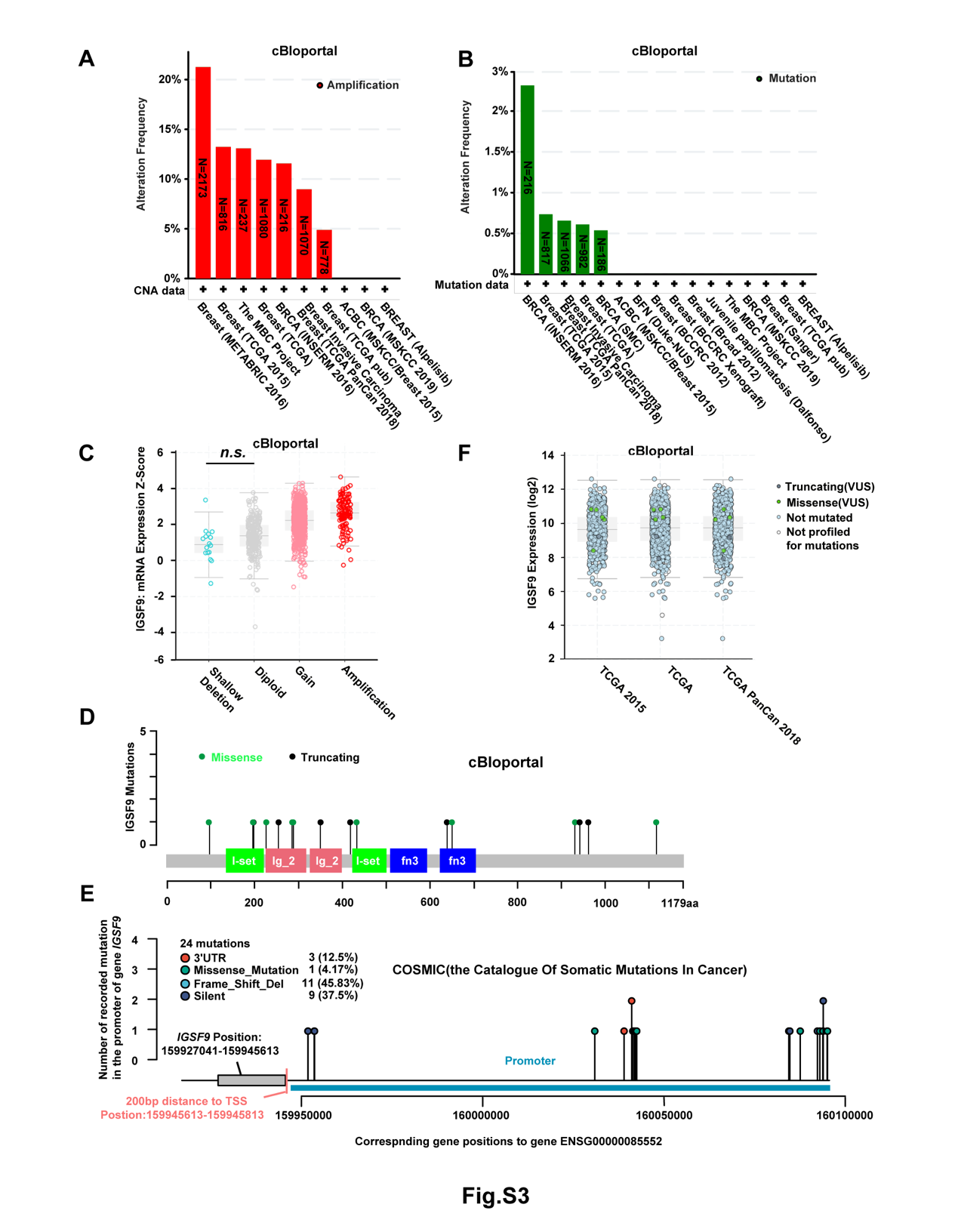
**

Figure S3. IGSF9 copy number and mutations don’t alter *IGSF9* mRNA levels

**A** Frequency of breast cancer *IGSF9* copy number alteration in different datasets (METABRIC; TCGA, 2015; MBC; TCGA; INSERM 2016; TCGA PanCan 2018; TCGA pub; MSKCC; Alpelisib) from cBioportal. Red, amplification. **B** Frequency of *IGSF9* mutation in breast cancer in different datasets (INSERM 2016; TCGA 2015; TCGA PanCan 2018; TCGA; SMC) from cBioportal. **C** *IGSF9* deletion is not correlated with decreased mRNA level. Box and whisker plot the correlation between mRNA levels and copy number alteration of *IGSF9* in breast cancer. **D** *IGSF9* mutation pattern in coding sequence. Green dot, missense mutation. Black dot, truncating mutation. mRNA of *IGSF9* mutation uniformly distributed in overall levels of breast cancer. **E** No mutation in the promoter of *IGSF9* based on COSMIC. Red dot, 3’UTR. Green dot, missense mutation. Light blue dot, frame-shift-deletion. Dark blue dot, silent. **F** Box and whisker plot of the correlation between mRNA levels and mutation of *IGSF9* in breast cancer. Green dot, missense mutation. Black dot, truncating mutation. Blue dot, non-mutation. White dot, not profiled mutation.

**
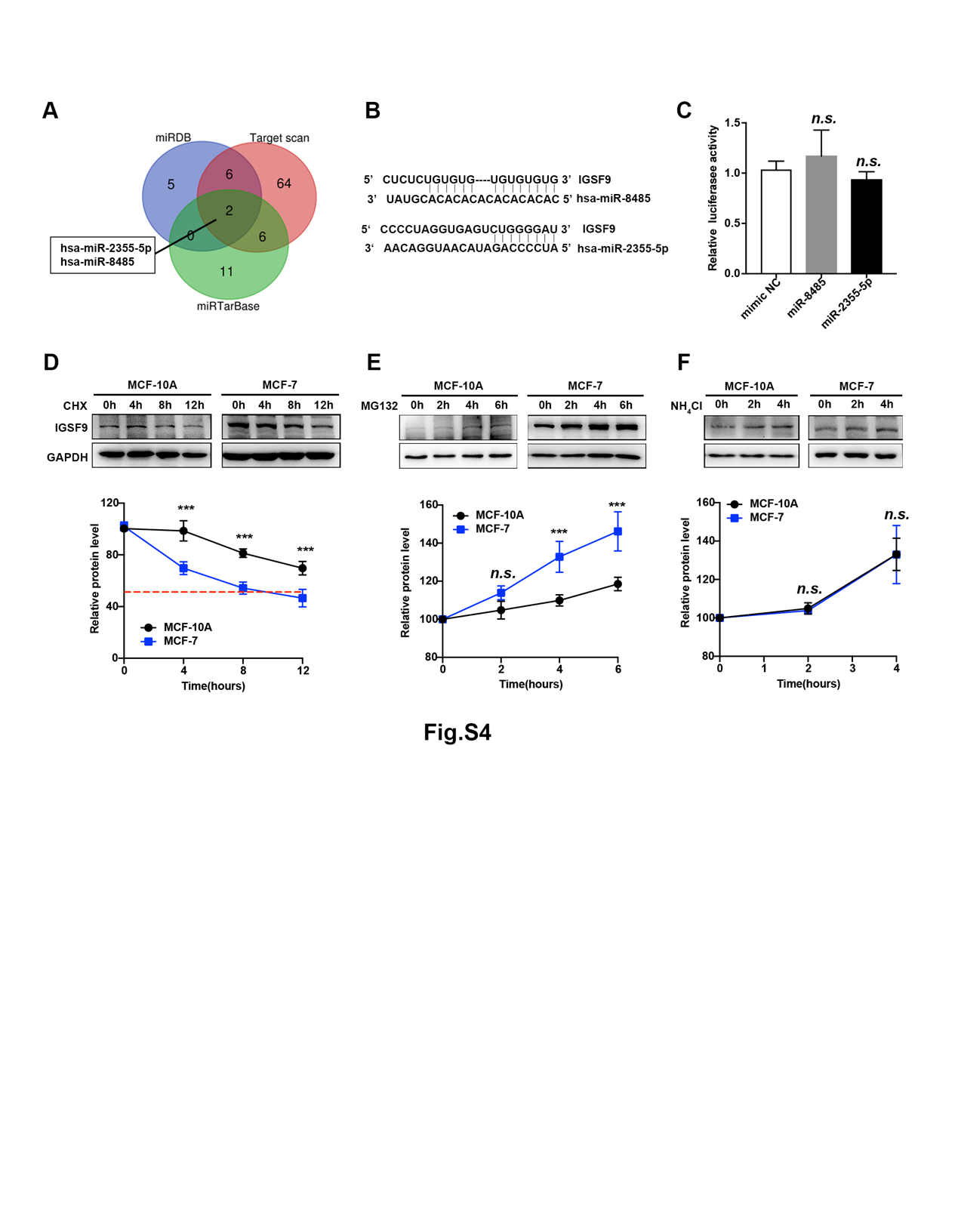
**

Figure S4. **IGSF9 protein stability is decreased in breast cancer cells**

**A** Schematic diagram of predicted 2 miRNAs for IGSF9 regulation in three bioinformatics algorithms. Each circle denotes one algorithm. **B** Predicted miR-8485 and miR-2355-5p target sequences in the 3’UTR of *IGSF9*. **C** miR-8485 and miR-2355-5p do not change luciferase activity of *IGSF9* 3’UTR. Error bars are presented as the mean ± SD. The Student's *t*-test was used. n.s. indicates no statistically significance. **D** MCF-7 showed shorter half-life of IGSF9 than MCF-10A. MCF-10A and MCF-7 cells were treated with or without 20 μM cycloheximide. Cells were harvested at indicated time. Cell lysates were subjected to western blot. GAPDH was used as a loading control. The quantitative data of IGSF9 protein level are represented in the lower panel. Data are represented as the mean ± SD. The Student’s *t*-test was used, ****P* **<** 0.001, n.s. indicates no statistically significance. **E** IGSF9 protein is degraded via proteasomal pathway. MCF-10A and MCF-7 cells were treated with or without 20 μM MG132. Cells were harvested at mentioned time points. Cell lysates were subjected to western blot. GAPDH was used as a loading control. The quantitative data of IGSF9 protein level are represented in the lower panel. Data are represented as the mean ± SD. The Student’s *t*-test was used, ****P* **<** 0.001, n.s. indicates no statistically significance. **F** Lysosome does not contribute to IGSF9 degradation. MCF-10A and MCF-7 cells were treated with or without 25 mM NH_4_Cl. Cells were harvested at mentioned time points. Cell lysates were subjected to western blot. GAPDH was used as a loading control. The quantitative data of IGSF9 protein level are represented in the lower panel. Data are represented as the mean ± SD. The Student’s *t*-test was used, ****P* **<** 0.001, n.s. indicates no statistically significance.


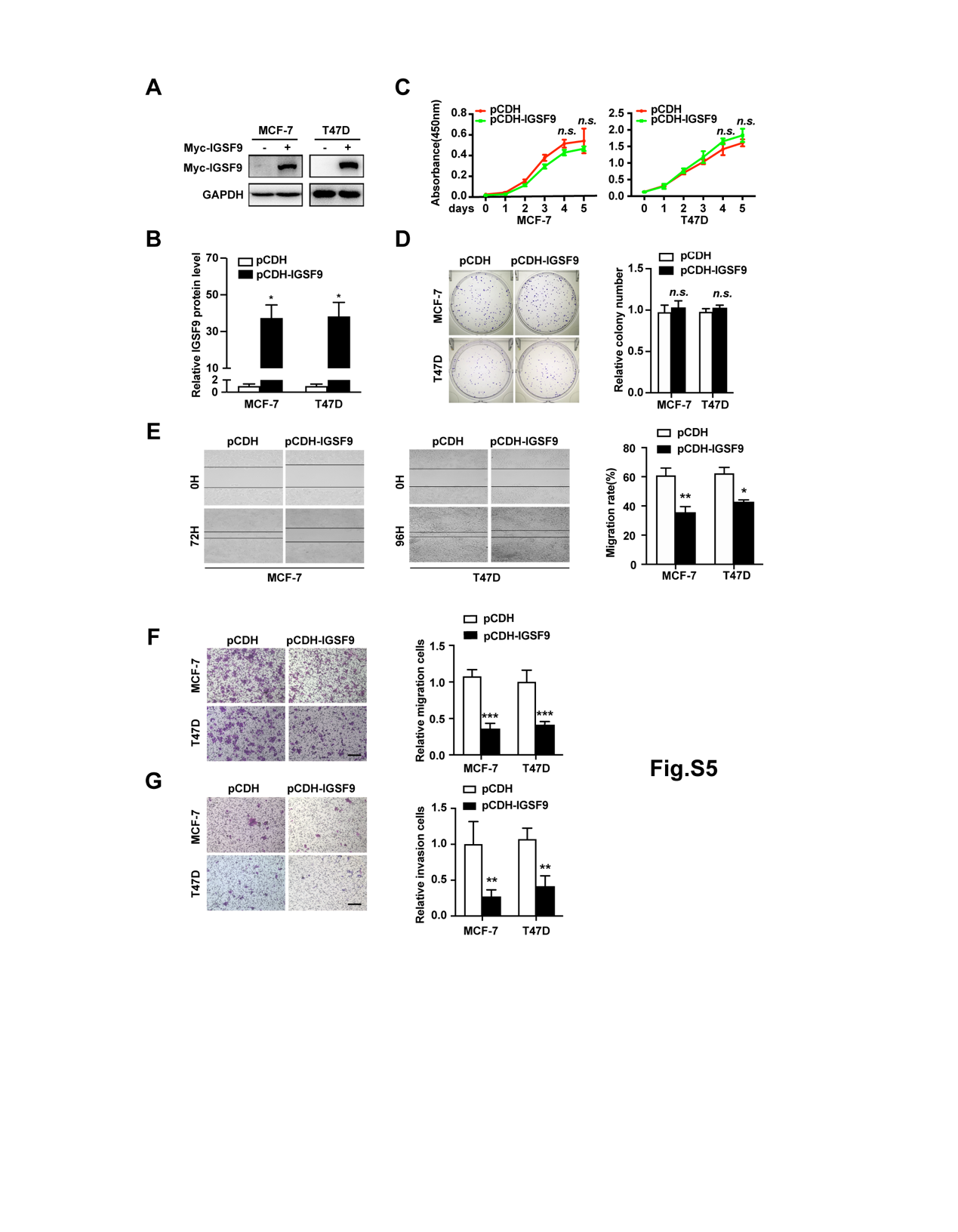


Figure S5. **IGSF9 inhibits breast cancer migration and invasion, but not proliferation**

**A-B** Over-expression of IGSF9 in MCF-7 and T47D cells. IGSF9 protein levels were detected by western blot (**A**) and normalized by GAPDH (**B**). Data are represented as the mean ± SD. The Student’s *t*-test was used, **P* **<**0.05. **C** IGSF9 does not affect breast cell proliferation. CCK-8 assays were performed with the stable cells mentioned above. Data are represented as the mean ± SD. n.s. indicates no statistically significance. **D** IGSF9 does not affect breast cancer cell colony formation. Relative colony numbers were counted and shown. Data are represented as the mean ± SD. **E** IGSF9 over-expression inhibits the migration of breast cancer cell lines. Wound-healing assays were conducted with referred breast cancer cells. Values were mean ± SD. The Student’s *t*-test was used, **P* **<**0.05, ***P* **<**0.01. **F-G** IGSF9 over-expression inhibits the migration (**F**) and invasion (**G**) of breast cancer cells. Transwell assays were used to detect the migration and invasion abilities of these stable cell lines. Quantification was performed by ImageJ. Scale bar, 100 μm. Data are represented as the mean ± SD. The Student’s *t*-test was used, ***P* **<**0.01, ****P* **<**0.001.

**Supplementary Table S1. Primers in this study.**

| Name | Sequences（5’-3’） |
| --- | --- |
| IGSF9 ChIP F | CTGCTGCCCCTCTTGCTG |
| IGSF9 ChIP R | GAGGCACGGCGAGGCGGGCGGGACC |
| GAPDH ChIP F | TACTAGCGGTTTTACGGGCG |
| GAPDH ChIP R | TCGAACAGGAGGAGCAGAGAGCGA |
| FAK (1-355) F | AAGGAAAAAAGCGGCCGCGCCACCATGGCAGCTGCTTACCTTGACCCCA |
| FAK (1-355) R | GGGGTACCTCAAGCATAATCTGGAACATCATATGGATACGAGGTTCCATTCACC |
| FAK (356-680) F | AAGGAAAAAAGCGGCCGCGCCACCATGCAGTCATTTATCA |
| FAK (356-680) R | GGGGTACCTCAAGCATAATCTGGAACATCATATGGATACAGGATTGTGCTGAGC |
| FAK (681-1052) F | AAGGAAAAAAGCGGCCGCGCCACCATGGAGGAAGAGAAGGCTCAG |
| FAK (681-1052) R | GGGGTACCTCAAGCATAATCTGGAACATCATATGGATAGTGTGGTCTCGTCTGC |
| IGSF9(1-493) F | GCTCTAGAGCCACCATGATTTGGTGTCTCCGTCTGACCG |
| IGSF9(1-493) R | CCCAAGCTTTCACAGATCCTCTTCAGAGATGAGTTTCTGCTCGGCTACAGCATTGCTGGCACTGCAT |
| IGSF9(494-736) F | GCTCTAGAGCCACCATGCGTGTGACCACTTCCACCAATGTAT |
| IGSF9(494-736) R | CCCAAGCTTTCACAGATCCTCTTCAGAGATGAGTTTCTGCTCCAATACAGGCTGGGGCAGGAGACCT |
| IGSF9(737-1179) F | GCTCTAGAGCCACCATGGCTGGTGTTGTGGGTGGAGTCTGCT |
| IGSF9(737-1179) R | CCCAAGCTTTCACAGATCCTCTTCAGAGATGAGTTTCTGCTCTAGCAGAGTAGCCTGTTCAGG |
| GST-FAK (1-355) F | CGGGATCCGCCACCATGGCAGCTGCTTACCTTGACCCCA |
| GST-FAK (1-355) R | CATGCCATGGTCACGAGGTTCCATTCACC |
| pGL3-3000 F | GGGGTACCATGCTTCTTTGTGCAGGGCTAT |
| pGL3-3000 R | CCGCTCGAGTCAATCTCCCTCCCCGCTGTTAGAATTG |
| pGL3-2000 F | GGGGTACCATGCCTTCCCAATGATTGGTAAAGATCC |
| pGL3-2000 R | CCGCTCGAGTCAGGATTGAGACCCAACCCAAACTTC |
| pGL3-1000 F | GGGGTACCATGAGCTCTGCCATAACCTA |
| pGL3-1000 R | CCGCTCGAGTCATACCTCCAGTACGCCCCCTCCCT |
| pGL3-1000s F | GGGGTACCATGAGCTCTGCCATAACCTAG |
| pGL3-1000s R | CCGCTCGAGTCAGTGTGCAGCTGAGGAAGGCTGTG |
| pGL3-617 F | GGGGTACCATGGGTGGGAGGCGCCTGGGCAAGATTT |
| pGL3-617 R | CCGCTCGAGTCAGGTCCCGGGCTCATCCGATCT |
| pGL3-450 F | GGGGTACCATGCGCCTGGTTCTCCTACCCCCA |
| pGL3-450 R | CCGCTCGAGTCAGGTCCGGCTCGCGCTCCAGGGCTGC |
| pGL3-283 F | GGGGTACCATGCTGCTGCCCCTCTTGCTGGCGGCGG |
| pGL3-283 R | CCGCTCGAGTCAGAGGCACGGCGAGGCGGGCGGGACC |
| pGL3-116 F | GGGGTACCATGTGGCGGCTCCAATTGGCCGCCGCGG |
| pGL3-116 R | CCGCTCGAGTCATACCTCCAGTACGCCCCCTCCCT |
| pGL3-283s F | GGGGTACCATGCTGCTGCCCCTCTTGCTG |
| pGL3-283s R | CCGCTCGAGTCACGTGGAAAGGCCGGTGG |
| pGL3-233 F | GGGGTACCATGCTTGGCTGTGCAGAGG |
| pGL3-233 R | CCGCTCGAGTCAGGCTGCACGTGGGCTCA |
| pGL3-183 F | GGGGTACCATGGAGCTCTTCCCTCCTGAAACTA |
| pGL3-183 R | CCGCTCGAGTCAGAGGCACGGCGAGGCGGGCG |
| △-163to-159 F | GGGGTACCGAGCTCTTCCCTCCTGAAACTCTCCCAGGCCGCTCAGGGGTCCCGCCCGCCTCGCCGTGCCTCCTCGAGCG |
| △-163to-159 R | CGCTCGAGGAGGCACGGCGAGGCGGGCGGGACCCCTGAGCGGCCTGGGAGAGTTTCAGGAGGGAAGAGCTCGGTACCCC |
| △-137to-131 F | GGGGTACCGAGCTCTTCCCTCCTGAAACTACAACTCCCAGGCCGCTCAGGGGTGCCTCGCCGTGCCTCCTCGAGCGG |
| △-137to-131 R | CCGCTCGAGGAGGCACGGCGAGGCACCCCTGAGCGGCCTGGGAGTTGTAGTTTCAGGAGGGAAGAGCTCGGTACCCC |
| p53 R175H F | AGGTTGTGAGGcacTGCCCCCACCATGAGCGC |
| p53 R175H R | GCAgtgCCTCACAACCTCCGTCATGTGCTGTG |
| p53 Y220C F | TGGTGGTGCCCtgtGAGCCGCCTGAGGTTGG |
| p53 Y220C R | CTCacaGGGCACCACCACACTATGTCGAAAAG |
| p53 G244D F | TGCATGGaCGGCATGAACCGGAGGCCCATCCT |
| p53 G244D R | TTCATGCCGtCCATGCAGGAACTGTTACACATG |
| p53 G245S F | TGCATGGGCagcATGAACCGGAGGCCCATCCT |
| p53 G245S R | TTCATgctGCCCATGCAGGAACTGTTACACAT |
| p53 R248Q F | ATGAACcagAGGCCCATCCTCACCATCATCAC |
| p53 R248Q R | ATGGGCCTctgGTTCATGCCGCCCATGCAGGA |
| p53 R249S F | ATGAACCGGagcCCCATCCTCACCATCATCACA |
| p53 R249S R | ATGGGgctCCGGTTCATGCCGCCCATGCAGGA |
| p53 R273H F | TGAGGTGcatGTTTGTGCCTGTCCTGGGAGAG |
| p53 R273H R | CACAAACatgCACCTCAAAGCTGTTCCGTCCC |
| p53 R282W F | AGAGACtggCGCACAGAGGAAGAGAATCTCCG |
| p53 R282W R | TCTGTGCGccaGTCTCTCCCAGGACAGGCA |
| qPCR Primer: IGSF9 F | TTGGATTCCTGCTTCCCATC |
| qPCR Primer: IGSF9 R | AATCGTCTTCAGGGATGTGCTGGTC |
| qPCR Primer: GAPDH F | CGGAGTCAACGGATTTGGTCGTAT |
| qPCR Primer: GAPDH R | AGCCTTCTCCATGGTGGTGAAGAC |

**Supplementary Table S2.** **Antibodies used in this study**

| Antibody | Cat. No. | Dilution | Company |
| --- | --- | --- | --- |
| IGSF9 | HPA037753 | 1:1000 (WB) 1:50 (IHC)  1:50 (IF) | Sigma-Aldrich |
| α-tubulin | 2144 | 1:2000 (WB) | CST |
| GAPDH | 5174 | 1:2000 (WB) | CST |
| p53 | 2524 | 1:2000 (WB) | CST |
| Myc-tag | 2276 | 1:2000 (WB) 1:200 (IP)  1:200 (IF) | CST |
| HA-tag | 3724 | 1:2000 (WB) 1:200 (IP) | CST |
| EMT marker | #9782 | 1:2000 (WB) 1;200 (IF) | CST |
| FAK | 71433 | 1:2000 (WB) 1:200 (IF) | CST |
| FAK-p397 | 3283 | 1:2000 (WB) | CST |
| AKT | 4685 | 1:2000 (WB) | CST |
| AKT-p473 | 4060 | 1:2000 (WB) | CST |
| AKT-p308 | 13038 | 1:2000 (WB) | CST |
| DAPI | ab228549 | 1:10000 (IF) | Abcam |
| Goat Anti-Rabbit IgG (H+L) | 111-035-003 | 1:10000 (WB)  1:200 (IHC) | Jackson |
| Goat Anti-Mouse IgG (H+L) | 115-035-003 | 1:10000 (WB)  1:200 (IHC) | Jackson |
| Goat Anti-Rabbit IgG (H+L) Alexa Fluor® 594 | 111-585-003 | 1:500 (IF) | Jackson |
| Goat Anti-Mouse IgG (H+L) Alexa Fluor® 488 | 111-545-003 | 1:500 (IF) | Jackson |

CST, Cell Signaling Technology (Beverly, MA, USA); Jackson, Jackson ImmunoResearch (West Grove, PA, USA); Abcam (CA, USA); Sigma-Aldrich (St. Louis, MO, USA).
